# Clinical efficacy of PPAR agonists in the treatment of nonalcoholic fatty liver disease

**DOI:** 10.3389/fphar.2026.1775516

**Published:** 2026-03-27

**Authors:** Xiaoling Li, Junjie Zhang, Bo Wei, Huan Tong

**Affiliations:** 1 Department of Gastroenterology and Hepatology, West China Hospital, Sichuan University, Chengdu, China; 2 West China School of Medicine, Sichuan University, Chengdu, China

**Keywords:** clinical efficacy, nonalcoholic fatty liver disease, nonalcoholic steatohepatitis, peroxisome proliferator-activated receptor, randomized controlled trial

## Abstract

Nonalcoholic fatty liver disease (NAFLD) has become a major public health concern. Peroxisome proliferator-activated receptors (PPARs) are nuclear receptor transcription factors. PPARs are categorized into three subtypes: PPARα, β/δ, and γ. Three subtypes of PPARs play crucial roles in lipid and glucose metabolism, inflammation and fibrosis. This review summarizes randomized controlled trials on the use of PPAR agonists in the treatment of NAFLD. PPARα and PPARβ/δ agonists control circulatory lipids well, but they do not yield enough liver benefits. PPARγ agonists, particularly pioglitazone, are recommended for NAFLD treatment. PPARα/γ agonists and PPARα/β/γ agonists are plausible in the treatment of NAFLD. More clinical studies are still in need to properly unveil the efficacy of PPAR agonists in the treatment of NAFLD.

## Introduction

The prevalence of nonalcoholic fatty liver disease (NAFLD) has increased globally in recent years, affecting 43% of overweight and obese children and increasing public health concerns (Ruan et al., 2025; Xiao et al., 2025). Middle-aged and elderly people remain the group with a high incidence of fatty liver disease, while the onset age is gradually becoming younger due to the increase in obesity and alcohol use (Pitisuttithum and Treeprasertsuk, 2022; Ginès et al., 2021). NAFLD pathogenesis involves a complex interplay of metabolic homeostasis, oxidative stress and inflammation, pivoting on insulin resistance and hepatic lipid accumulation (European Association for the Study of the Liver, European Association for the Study of Diabetes, European Association for the Study of Obesity, 2016). Male gender, higher body mass index, dyslipidemia and elevated levels of fasting blood glucose, etc., are potential risk factors for NAFLD (Xiao et al., 2025). Type 2 diabetes mellitus (T2DM) and NAFLD are closely linked to each other as NAFLD is a leading chronic disorder linked to diabetes mellitus and its cardiovascular and renal complications while individuals with DM are at high risk for developing NAFLD, and their bilateral interactions significantly increase the risk of both liver-related and extrahepatic adverse outcomes (Chinese Society of Health Management, Liver and Gallbladder Diseases Branch of China Association of Chinese Medicine, Liver Health Consortium in China CHESSLiver and Gallbladder Diseases Branch of China Association of Chinese MedicineLiver Health Consortium in China CHESS, 2026; Yang et al., 2026). NAFLD and nonalcoholic steatohepatitis (NASH) have been renamed as metabolic dysfunction-associated steatotic liver disease (MASLD) and metabolic dysfunction associated steatohepatitis (MASH) recently, however, most of the studies included in this review used the terms NAFLD and NASH. To maintain consistency, NAFLD and NASH are used in the review hereafter, and NAFLD in this review generally equals MASLD, as well as that NASH generally equals MASH.

The fundamental treatment option for NAFLD is lifestyle change. However, lifestyle change faces many challenges, as only 10% of patients lose more than 10% of their body weight after their lifestyle changes (Alkhouri and Scott, 2018). For patients with more severe MASLD, namely, MASH, the sole use of lifestyle intervention is not enough for treatment. Therefore, there is an intensive and increasing need for medications to treat MASLD.

An increasing number of investigations into new molecular targets of NAFLD have shed light on the treatment of NAFLD. Peroxisome proliferator-activated receptors (PPARs) are one hotspot of NAFLD. Since the 1960s, scholars have discovered that many compounds can induce the proliferation of peroxisomes (Islinger et al., 2018). In 1990, a study found that PPAR is a protein residing in liver of mice (Issemann and Green, 1990). This review focuses on basic information on PPARs and related randomized clinical trials (RCTs) on the use of PPAR agonists in the treatment of MASLD.

### Peroxisome proliferator-activated receptors

**FIGURE 1:**
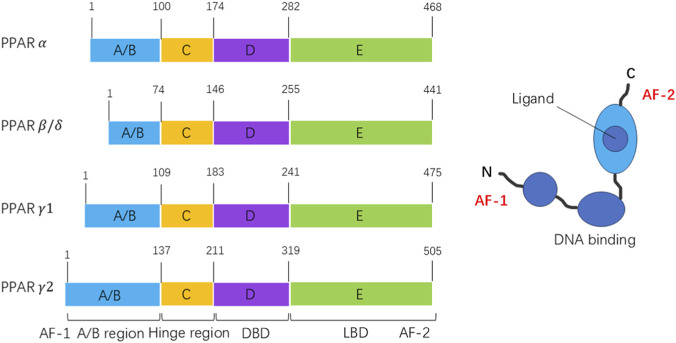
Structures of PPAR subtypes.

PPARs regulate the expression of genes responsible for energy metabolism, cellular development, inflammation, and differentiation (Qiu et al., 2023). PPARs have four functional domains: an N-terminal activation function domain (AF-1), a DNA binding domain (DBD), a hinge domain and a C-terminal ligand-binding domain (LBD) (Figure 1) (Barlaka et al., 2016). The PPAR family includes three subtypes: PPARα, PPARβ/δ, and PPARγ. These three subtypes of PPARs have distinct tissue distributions and different biological functions (Lefere et al., 2020). Upon ligand binding, PPARs combine with retinoid X receptors (RXRs) to form peroxisome proliferator response elements (PPREs) in the promoter regions of target genes, and thereafter modulate the expression of genes responsible for energy metabolism, cellular development, inflammation and differentiation. There have been plenty of studies on PPARs in liver diseases (Higashi et al., 2017).

PPARα is expressed mostly in the liver and brown adipose tissue. Palmitic acid and leukotriene B4 are common ligands and PPARα regulates the expression of genes such as lipoprotein lipase (LPL), Apo CIII, and Apo AI, reducing Apo CIII transcription and increasing the production and activity of LPL and Apo AI (Luo et al., 2023). PPARα also promotes the uptake of fatty acids by the liver, inhibits the synthesis of TG, and reduces lipoproteins containing TG. In addition, PPARα promotes mitochondrial β-oxidation and reduces oxidative stress damage and lipid accumulation in the liver (Wang et al., 2024), and PPARα also inhibits liver fibrosis by its transrepressive activity (Pawlak et al., 2014).

PPARβ/δ is widely expressed in various tissues and plays an important role in the regulation of carbohydrate and lipid metabolism in the liver (Zarei et al., 2021). Natural ligands for PPARβ/δ include fatty acid and retinoic acid. PPARβ/δ is reported to alter the expression of genes related to glucose metabolism and improve insulin sensitivity (Zarei et al., 2019). It enhances glucose transporter 4 (GLUT4) expression, improving insulin sensitivity and glucose uptake. PPARβ/δ activation reduces expression of proinflammatory cytokines such as TNFα, IL-1β and CCL2 (Shan et al., 2008), whereas its effect is equivocal in liver fibrosis (Kostadinova et al., 2012; Iwaisako et al., 2012).

PPARγ is highly expressed in white adipose tissue, and plays as a key regulator of adipocyte differentiation and lipid storage. The activation of PPARγ can upregulate the expression of fatty acid binding protein 4 and lipoprotein lipase, promoting the uptake and storage of fatty acids (Lei et al., 2022). By increasing lipid storage in the adipose tissue, PPARγ inhibits peripheral fat decomposition and ectopic deposition in the liver, thereby decreasing hepatic steatosis. PPARγ also improves insulin sensitivity through its effects on adipose tissue (Lee et al., 2021). In addition, PPARγ promotes intracellular glutamine metabolism to induce macrophage polarization to the anti-inflammatory M2 phenotype (Bouhlel et al., 2007; Nelson et al., 2018). PPARγ agonist also reduces hepatic stellate cell activation (Li et al., 2015).

### PPARα agonists and NAFLD

#### Fenofibrate

Fenofibrate is a PPARα agonist commonly used to lower triglyceride levels. Its efficacy in the management of NAFLD has been investigated in several RCTs. In one RCT including obese NAFLD patients diagnosed by MRI, fenofibrate significantly reduced plasma triglyceride levels and very low-density lipoprotein, but it did not significantly affect insulin sensitivity or intrahepatic triglyceride content (Fabbrini et al., 2010). These findings are consistent with another RCT including NAFLD patients with hypertriglyceridemia, and the most common adverse events of fenofibrate occurred in the gastrointestinal system (Oscarsson et al., 2018). Based on the accumulated data, fenofibrate could not substantially ameliorate NAFLD despite that it control serum lipids well.

#### Pemafibrate

Pemafibrate (K-877) is a novel selective PPARα modulator approved for the treatment of dyslipidemia in Japan. Pemafibrate exhibits more potency and selectivity than does PPARα, and it presents a more favorable renal and hepatic safety profile than does fenofibrate (Ishibashi et al., 2018). In an RCT, pemafibrate did not significantly reduce the liver fat content of NAFLD patients, but it led to a 6.2% reduction in liver stiffness at week 72. It also diminished ALT, GGT, ALP and lipid levels. The treatment was well tolerated without a significant increase in adverse events (Nakajima et al., 2021). This study implies pemafibrate may perform better than fenofibrate in treating NAFLD, and it emerges as a potential therapeutic agent for NAFLD.

### PPARβ/δ agonists and NAFLD

Seladelpar (MBX-8025) is an innovative selective PPARβ/δ agonist, and its efficacy was evaluated in an RCT enrolling biopsy-confirmed NASH. Seladelpar at different dosages (10 mg/d, 20 mg/d or 50 mg/d) significantly improved liver enzymes, lipid profiles, and serum inflammatory marker levels. However, the liver fat content did not differ between seladelpar and placebo groups (Jang et al., 2020). The trial was halted in November 2019 because of atypical histological findings, such as interface hepatitis. However, these findings were not considered consistent with seladelpar-induced liver injury and a subsequent expert review revealed that these histological features were common in patients with advanced NASH, which may have been previously underrecognized (Dhingra et al., 2022). Similar to fenofibrate, although seladelpar improves the lipid profiles of patients with NASH, its therapeutic efficacy in treating NASH remains unsatisfactory.

### PPARγ agonists and NAFLD

#### Pioglitazone

Pioglitazone, a second-generation thiazolidinedione derivative, is a PPARγ agonist that was initially developed by Japanese and American companies in the 1980s and was approved for market in 1999. It gradually entered Europe and China and has been widely used as an insulin sensitizer for T2DM (Waugh et al., 2006). Although side effects limit its utility, its value has been recognized globally and it was approved for the treatment of T2DM, especially for patients with NASH (Lehrke and Lazar, 2005). Beyond its established efficacy in treating T2DM and NASH, pioglitazone has demonstrated significant cardiovascular risk reduction in high-risk populations, which is attributed mainly to its anti-atherogenic and anti-inflammatory properties, and has shown metabolic improvement in polycystic ovary syndrome (Dorm et al., 2005; Zhao et al., 2024). In addition to its impact on glucose and lipid metabolism, pioglitazone exerts diverse effects relevant to NASH pathogenesis, including modulation of adipose tissue function, redistribution of liver lipids, suppression of hepatic inflammation, and indirect antifibrotic signaling via adiponectin upregulation (Shaaban et al., 2022; de Mendonça et al., 2019) Recently, the mitochondrial pyruvate carrier (MPC) has been identified as a previously unrecognized target of pioglitazone, suggesting additional mechanisms beyond classical nuclear PPARγ activation (Divakaruni et al., 2013). Pioglitazone has been adequately investigated for the treatment of NAFLD/NASH until now.

Diabetes is an important comorbidity of NAFLD, and diabetes can worsen its progression. Therefore, the co-existence with NAFLD and diabetes has become a hotspot in NAFLD treatment. In two double-blind, placebo-controlled RCTs, pioglitazone markedly mitigated steatosis, ballooning necrosis, and inflammation of the liver in biopsy-confirmed NASH patients with either impaired glucose tolerance or type 2 diabetes, although liver fibrosis did not improve (Belfort et al., 2006). This finding was further supported by another RCT in biopsy-proven NASH patients, and pioglitazone led to 41% more patients with liver histology alleviation and 32% more patients with NASH resolution, comparing with placebo (Cusi et al., 2016; Adil et al., 2024). Similarly, an extra RCT showed that pioglitazone could increase patients with ≥2-point NAS reduction by 35% in biopsy-proven NASH and T2DM patients, whereas fibrosis could not be further improved (Bril et al., 2019). Additionally, in Chinese population with T2DM and NAFLD, the fixed-dose combination of pioglitazone and metformin gained a greater reduction in liver fat content and γ-GT decrease, comparing with metformin alone (Jianfang et al., 2023).

Pioglitazone was also evaluated in NASH patients without diabetes. In the PIVENS trial, a total of 247 adults with biopsy-confirmed NASH but without diabetes and comprehensive histological improvement were not obtained by pioglitazone. However, the resolution rate of steatohepatitis in the pioglitazone group was 25% higher than that in the placebo group (Sanyal et al., 2010). Similar to a western world study, an RCT conducted in Taiwan Province of China revealed that 46.7% of NASH patients in the pioglitazone group achieved NASH improvement without worsened fibrosis, which was 35.6% higher than that in the placebo group, despite no significant improvement in fibrosis or hepatocyte ballooning (Huang et al., 2021).

Based on the clinical trials described above, pioglitazone offers robust metabolic and anti-steatohepatitis efficacy, but its antifibrotic benefit is limited. Currently, clinical practice guidelines recommend pioglitazone in MASH (Adil et al., 2024). Notably, because of discordant role in the adipose tissue and liver, pioglitazone was associated with modest weight gain (+5.7 kg) while liver steatosis was contained (Bril et al., 2019). In addition, other PPARγ-related adverse effects, including peripheral edema, and increased fracture risk should also be carefully monitored.

#### Rosiglitazone

Similar to pioglitazone, rosiglitazone is a thiazolidinedione and selective PPAR-γ agonist, and it is primarily utilized to improve insulin sensitivity in T2DM. One RCT investigated the efficacy of rosiglitazone in biopsy-proven NASH. Rosiglitazone mitigated hepatic steatosis in 43% more patients and normalized serum transaminases in more 25% patients, comparing with placebo. Nevertheless, no significant changes were observed in liver fibrosis or inflammation. Even after an additional two-year extension, extended treatment with rosiglitazone did not improve the NAS score, ballooning, inflammation, or fibrosis (Ratziu et al., 2010). The main adverse events were modest weight gain and mild anemia without hepatic toxicity. However, its clinical use is restricted in many countries due to concerns about cardiovascular risk.

#### MSDC-0602K

MSDC-0602K is a second-generation TZD designed to minimize directly binding to PPARγ while retaining modulation of mitochondria pyruvate carrier, thereby improving insulin sensitivity independent of classical nuclear PPARγ signaling pathway. As a result, the risk of PPARγ-mediated adverse effects, such as fluid retention and bone fractures, was apparently avoided.

MSDC-0602K was studied in patients with biopsy-confirmed NASH and fibrosis stages F1–F3. This RCT suggested that histological improvement (≥2-point NAS reduction with a ≥1-point decrease in ballooning or lobular inflammation and no fibrosis worsening), NAS improvement without worsening fibrosis, NASH resolution and fibrosis reduction were not reached by MSDC-0602K. Despite the lack of histological efficacy, MSDC-0602K, particularly at higher doses, led to significant reductions in fasting glucose, HbA1c, HOMA-IR, insulin, and liver enzymes (ALT, AST) without increasing adverse events such as edema, hypoglycemia, or fractures. Weight gain was observed but was not associated with worsening insulin resistance (Harrison et al., 2020).

#### PXL065

Unlike pioglitazone, which exists as a racemic mixture of rapidly interconverting R- and S-enantiomers, PXL065 is a deuterium-stabilized R-pioglitazone. It lacks PPARγ activity attributed to the S-enantiomer but maintains non-genomic mechanisms, thereby inhibiting potential PPARγ-driven side effects such as weight gain and edema while preserving therapeutic efficacy in the treatment of NASH. The efficacy of PXL065 in biopsy-confirmed NASH and fibrosis stages F1–F3 was assessed in DESTINY-1 trial. PXL065 dosage was evaluated at 7.5 mg, 15 mg, and 22.5 mg daily. A significant reduction in liver fat content was reached in all PXL065 groups, ranging from −21% to −25%. Patient numbers of ≥1-stage fibrosis improvement and ≥2-point NAS reduction without fibrosis worsening were 33% and 20% higher in 15 mg PXL065 group, comparing with placebo group, respectively. PXL065 also displayed dose-dependent improvements in HbA1c, insulin sensitivity (HOMA-IR, Adipo-IR), and adiponectin levels. Importantly, PXL065 was well tolerated, with no evidence of dose-dependent weight gain, edema, and anemia (Harrison et al., 2023). Therefore, PXL065 possesses metabolic and hepatic benefits in NAFLD avoiding classical PPAR γ-mediated adverse effects.

### Dual PPARα/γ agonist and NAFLD

Saroglitazar is a dual PPARα and γ agonist, and it activates PPARα potently and PPARγ modestly. Ideally, it corporates the advantages of PPARα agonist and PPARγ agonist. Saroglitazar was initially approved in India for the treatment of diabetic dyslipidemia and subsequently received approval for NASH treatment in 2020 (Kaul et al., 2019). Saroglitazar was investigated in an RCT to optimize its dosage in MAFLD. Imaging or biopsy confirmed NAFLD patients received saroglitazar at different dosages (1 mg/d, 2 mg/d and 4 mg/d). All saroglitazar groups exhibited significant dose-dependent reductions in ALT at week 16 from −25.5% (1 mg/d) to −45.8% (4 mg/d). It is impressive that saroglitazar of 4 mg/d group presented decrease liver fat content by 19.7% and 30% recipients at this dosage reached 30% reduction in liver fat content. Saroglitazar of 4 mg/d also obtained the best improvements in liver fibrosis (enhanced liver fibrosis LS-0.22, liver stiffness −1.9 KPa), and lipid profiles (triglyceride LS-68.7, very-low-density lipoprotein LS-7.4). However, saroglitazar did not improve body weight, glucose, HbA1c, as well as insulin levels. The most common adverse effects were diarrhea, cough, abdominal pain and bronchitis with low occurrences (<3.85%) (Gawrieh et al., 2021).

For the more severe form of NAFLD, NASH was studied with saroglitazar in another RCT, and the performance of saroglitazar was similar to that of NAFLD. Saroglitazar markedly mitigated steatosis and hepatocyte ballooning by −0.50∼ -0.71 and −0.83∼-0.86 without liver fibrosis alleviation. Saroglitazar improved the lipid profile (low-density lipoprotein and cholesterol), whereas it had tiny glycemic parameters. The clinical efficacy presented as the dosage-dependent manner (Siddiqui et al., 2021). Saroglitazar appears to function well in liver and circulation lipid metabolism, but poorly in liver fibrosis and glucose metabolism. It is generally safe, but its adverse effects on the gastrointestinal and respiratory systems still need to be considered with caution.

### Dual PPAR α/δ agonist and NAFLD

Elafibranor (GFT505) is a dual peroxisome proliferator-activated receptor α/δ agonist developed for the treatment of NASH and other metabolic disorders. An RCT in which elafibranor was used to treat histologically confirmed NASH investigated the efficacy of elafibranor. Elafibranor at 120 mg/d significantly improved NASH resolution without worsening fibrosis (19% vs. placebo 12%), with more responders among patients with a baseline NAS ≥4 (20% vs. placebo 11%). Elafibranor also improved liver enzymes, lipid profiles, glucose metabolism, and markers of systemic inflammation. The treatment was well tolerated, with no significant weight gain or cardiac events. In terms of adverse effects, a mild reversible increase in serum creatinine was observed (Ratziu et al., 2016).

However, when the treatment duration of elafibranor ranged from 52 weeks to 72 weeks, it failed to meet the primary endpoint of NASH resolution without fibrosis progression in the interim analysis of the RESOLVE-IT trial. This outcome was starkly in contrast to that of earlier trial. Possible explanations for this controversy include a reliance on *post hoc* endpoints, modest histological efficacy, and inadequate antifibrotic activity. Anyway, the studies of elafibranor for NASH treatment were terminated (Drenth and Schattenberg, 2020; Goyal et al., 2023).

### Pan-PPAR agonists and NAFLD

Lanifibranor is the first pan‐peroxisome proliferator‐activated receptor agonist characterized by stronger activation of PPARα and δ and relatively weaker activation of PPARγ. A lanifibranor dosage of 1,200 mg/d rendered more NASH decrease of at least 2 points in the SAF-A score (55% vs. placebo 33%). Both 120 mg/d and 800 mg/d lanifibranors resulted in a greater percentage of patients with resolution of NASH and regression of fibrosis (1,200 mg/d 49%, 800 mg/d 39% vs. placebo 22%). Lanifibranor improved the serum aminotransferase levels, markers of glucose metabolism, plasma lipid profiles and biomarkers of inflammation and fibrosis. However, adverse events such as nausea, diarrhea, and weight gain occurred more frequently in the lanifibranor groups (Francque et al., 2021).

Complementing this, another RCT investigated the efficacy of lanifibranor in patient with NAFLD and T2DM. Lanifibranor exhibited a 32% more reduction in intrahepatic triglyceride (IHTG) content, with a greater proportion achieving a ≥30% reduction in IHTG. Moreover, lanifibranor improved hepatic and peripheral insulin sensitivity, decreased plasma aminotransferases and HbA1c, and increased plasma adiponectin and HDL-C levels. Treatment with lanifibranor is generally well tolerated with slight weight gain (+2.7% vs. placebo −1.0%) (Barb et al., 2025). Overall, lanifibranor treatment is associated with sufficient histological efficacy while maintaining long-term safety and tolerability.

## Conclusion and perspectives

Benefiting the deeper understanding into the knowledge of structure and function of PPARs, PPARs have become a promising therapeutic targets for NAFLD over the past 2 decades. Sequential RCTs were conducted to study the efficacy of various PPAR agonists in NAFLD (Table 1). As for single PPAR agonists, PPARα and PPARβ/δ agonists control circulatory lipids well, but they do not yield enough liver benefits. PPARγ agonists, particularly pioglitazone, are the most validated options for NAFLD treatment. However, the side effects of PPARγ agonists, such as weight gain, edema and increased fracture risk, should raise caution from the practitioners when prescribing it. The next-generation agents PPARγ agonists would be a promising solution for minimizing adverse effects without compromising its efficacy. Although every subtype of PPAR has been shown to improve metabolism, inflammation and fibrosis of the liver in preclinical studies, however, RCTs suggest that only PPARγ agonists could be effective at treating NAFLD. That might be because NAFLD involves a complex network of mechanisms, and the activation of a single PPAR might incur the compensation driven by other molecules.

**TABLE 1:** PPAR agonists in randomized controlled clinical trials against NAFLD/NASH.

Drug name	Reference DOI	Participants	Intervention	Primary outcomes	Main adverse effect
Fenofibrate PPARα agonist	10.1210/jc.2009-2622	Obese subjects with NAFLD	Group 1: placebo for 8 weeks (n = 9) Group 2: Fenofibrate 200 mg/d for 8 weeks (n = 9) Group 3: ER niacin (Niaspan) for 16 weeks (titrated from 500 mg/wk to final dose of 2000 mg/wk during the first 3 weeks) (n = 9)	Changes in IHTG content: No significant in all groups Lipid: Significant decrease in plasma TG, VLDL-TG, and VLDL-apolipoprotein B concentrations in group 2 and group 3 Insulin sensitivity: Significant reduction in hepatic, adipose tissue, and muscle insulin sensitivity in group 3	Not mentioned
	10.1016/j.jacl.2018.08.003	Overweight or obese patients with hypertriglyceridemia and NAFLD	Group 1: 200 mg fenofibrate for 12 weeks (n = 27) Group 2: 4 g OM-3CA for 12 weeks (n = 25) Group 3: placebo for 12 weeks (n = 26)	Changes in liver PDFF: No significant +17%, −2% vs. +4%, all *P > 0.05*. (group 1, group 2 vs. group 3)	Diarrhea, abdominal pain and flatulence
Pemafibrate PPARα agonist	10.1111/apt.16596	Patients with NAFLD defined as liver fat content of ≥10% by MRI-PDFF	Group 1: pemafibrate 0.2 mg, twice daily for 72 weeks (n = 58) Group 2: placebo twice daily for 72 weeks (n = 60)	The percentage change in MRI‐PDFF from baseline to week 24: No significant, −5.3% vs. −4.2%, *P* = 0.85. (group 1 vs. group 2)	Blood creatinine phosphokinase increase, glycosylated hemoglobin increase, bronchitis, rash, cough, back pain, ligament sprain and diarrhea
Seladelpar PPARδ (PPARβ) agonist	10.1038/s41575-020-00366-5	Patients with histologically confirmed NASH	Group 1: seladelpar 10 mg/day for 52 weeks (n = 53) Group 2: seladelpar 20 mg/day for 52 weeks (n = 51) Group 3: seladelpar 50 mg/day for 52 weeks (n = 50) Group 4: placebo for 52 weeks (n = 27)	Reduction in liver fat content measured by MRI-PDFF: No significant, −9.76, −14.24, −13.00 vs. −20.78, all *P* > 0.05. (group 1, group 2,group 3 vs. group 4)	Abdominal pain, constipation, diarrhea, nausea, vomiting, fatigue, dizziness, headache and rash
Pioglitazone; PPARγ agonist	10.1056/NEJMoa060326	Patients with liver biopsy–confirmed NASH and impaired glucose tolerance or T2DM	Group 1: a hypocaloric diet plus pioglitazone 45 mg daily for 6 months (n = 26) Group 2: a hypocaloric diet plus placebo for 6 months (n = 21)	Liver histology Improvements in steatosis, ballooning necrosis and inflammation: Significant. (group 1 vs. group 2) Reduction in hepatic fat content: Significant, −54% vs. 0, *P* < 0.001. (group 1 vs. group 2)	Fatigue, mild lower-extremity edema, modest weight gain and increase body fat
	10.7326/M15-1774	Patients with histologically confirmed NASH and either prediabetes or T2DM	Group 1: pioglitazone (30 mg/d, titrated after 2 months to 45 mg/d) for 18 months (n = 50) Group 2: placebo for 18 months (n = 51)	Reduction of at least 2 points in the nonalcoholic fatty liver disease activity score in 2 histologic categories without worsening of fibrosis: Significant, 58% vs. 17%, *P* < 0.001. (group 1 vs*.* group 2)	Weight gain
	10.2337/dc19-0167	Patients with histologically confirmed NASH and T2DM	Group 1: vitamin E 400 IU b.i.d. for 18 months (n = 36) Group 2: vitamin E 400 IU b.i.d. plus pioglitazone 45 mg/day for 18 months (n = 37) Group 3: placebo for 18 months (n = 32)	Two-point reduction in the nonalcoholic fatty liver disease activity score from two different parameters, without worsening of fibrosis: Significant, 54% vs. 19%, *P* = 0.003 (group2 vs. group 3); No significant, 31% vs. 19%, *P* = 0.26 (group 1 vs. group 3)	Peripheral edema and weight gain
​	10.1155/2023/2044090	NAFLD patients with T2DM	Group 1: pioglitazone hydrochloride and metformin hydrochloride for 24 weeks (n = 60) Group 2: metformin hydrochloride for 24 weeks (n = 60)	Improvements in liver fat content: Significant. (group 1 vs. group 2) Decrease in *γ*-GT: Significant. (group 1 vs. group 2) Improvements in ALT, AST and insulin resistance index: No significant. (group 1 vs. group 2)	Increase waist circumference
	10.1097/MD.0000000000040356	NAFLD patients diagnosed via abdominal ultrasonography with T2DM	Group 1: ertugliflozin 15 mg daily for 24 weeks (n = 65) Group 2: pioglitazone 30 mg daily for 24 weeks (n = 65) Group 3: placebo for 24 weeks (n = 65)	Reduction in liver fat content: Significant in group 1 and group 2	Weight gain
	10.1056/NEJMoa0907929	Adults with liver biopsy–confirmed NASH and without diabetes	Group 1: pioglitazone 30 mg daily for 96 weeks (n = 80) Group 2: vitamin E 800 IU daily for 96 weeks (n = 84) Group 3: placebo for 96 weeks (n = 83)	Improvement in composite histologic findings (P < 0.025): Significant 43% vs. 19%, *P* = 0.001 (group 2 vs. group 3); No significant, 34% vs. 19%, *P* = 0.04 (group 1 vs. group 3)	Weight gain
	10.1007/s12072-021-10242-2	Taiwanese histologically confirmed NASH patients	Group 1: pioglitazone 30 mg/day for 6 months (n = 43) Group 2: placebo for 6 months (n = 47)	The efficacy of pioglitazone in reducing inflammation and liver fat at EOT: Both significant in group 1. (0.97 ± 0.56 to 0.63 ± 0.49, *P* = 0.002; 20.2% ± 9.0% to 14.3% ± 6.9%, *P* < 0.0001)	Insomnia and anxiety
Rosiglitazone; PPARγ agonist	10.1002/hep.23270	Patients with histologically proven NASH	Group 1: rosiglitazone (4 mg/day for the first month and 8 mg/day thereafter) for 1 year (n = 32) Group 2: placebo for 1 year (n = 31)	A reduction in steatosis >30% between baseline and EOT: Significant, 47% vs. 16%, *P* = 0.014. (group 1 vs. group 2)	Modest weight gain, peripheral edema, painful swollen legs and mild anemia
MSDC-0602K; PPARγ agonist	10.1016/j.jhep.2019.10.023	Patients with (biopsy-confirmed and NAS≥4) NASH and fibrosis (F1-F3)	Group 1: MSDC-0602K 62.5 mg daily for 52 weeks (n = 99) Group 2: MSDC-0602K 125 mg daily for 52 weeks (n = 98) Group 3: MSDC-0602K 250 mg daily for 52 weeks (n = 101) Group 4: placebo for 52 weeks (n = 94)	Hepatic histological improvement of ≥2 points in NAS with a ≥1-point reduction in either ballooning or lobular inflammation and no increase in fibrosis stage at 12 months: No significant, 29.8%, 32.9%, 39.5% vs. 29.7%, all *P >* 0.05. (group 1,2,3 vs. group 4)	Weight gain
PXL065 PPARγ agonist	10.1016/j.jhep.2023.02.004	Patients (≥8% liver fat, NAS ≥4, F1–F3)	Group 1: PXL065 7.5 mg daily for 36 weeks (n = 25) Group 2: PXL065 15 mg daily for 36 weeks (n = 32) Group 3: PXL065 22.5 mg daily for 36 weeks (n = 30) Group 4: placebo for 36 weeks (n = 30)	Relative change in the percentage of LFC from baseline to week 36: Significant, −21 to −25% vs. 2.4%, *P =* 0.008–0.02. (group 1,2,3 vs. group 4)	Modest weight gain
Saroglitazar PPARα/γ agonist	10.1002/hep.31843	Patients with NAFLD/NASH established either by imaging or liver biopsy	Group 1: saroglitazar 1 mg for 16 weeks (n = 26) Group 2: saroglitazar 2 mg for 16 weeks (n = 25) Group 3: saroglitazar 4 mg for 16 weeks (n = 27) Group 4: placebo for 16 weeks (n = 28)	Percentage change from baseline in ALT levels at Week 16: Significant dose-dependent reduction, −25.5%, −27.7%, −45.8% vs. 3.4%, all *P* < 0.05. (group 1,2,3 vs. group 4)	Diarrhea, cough, abdominal pain and bronchitis
​	10.1016/j.cgh.2020.10.051	Adult patients with biopsy-confirmed NASH	Group 1: saroglitazar magnesium 2 mg for 24 weeks (n = 6) Group 2: saroglitazar magnesium 4 mg for 24 weeks (n = 7) Group 3: placebo for 24 weeks (n = 3)	Change from baseline in NAS at week 24 (ΔNAS): No significant, −1.5 ± 0.84, −1.9 ± 1.57 vs. −1.3 ± 0.58, all *P >* 0.05. (group 1,2 vs. group 3)	Bronchitis, asthma and appendicitis
Elafibranor PPARα/δ agonist	10.1053/j.gastro.2016.01.038	Patients with NASH without cirrhosis	Group 1: elafibranor 80 mg for 52 weeks (n = 93) Group 2: elafibranor 120 mg for 52 weeks (n = 91) Group 3: placebo for 52 weeks (n = 92)	Resolution of NASH without fibrosis worsening, using modified definitions: Significant, 19% vs. 12%, *P* = 0 0.045 (group 2 vs. group 3); No significant, 13% vs. 12%, *P* = 0.8 (group 1 vs. group 3)	A mild and reversible increase in serum creatinine
	10.1080/13543784.2020.1839888	Patients with NASH	Group 1: elafibranor 120 mg for 72 weeks (n = 1,437) Group 2: placebo for 72 weeks (n = 720)	Resolution of nonalcoholic steatohepatitis without worsening of fibrosis: No significant, 19.2% vs. 14.7%, *P* = 0 0.0659. (group 1 vs. group 2)	Abdominal pain, diarrhea, nausea, vomiting, fatigue and arthralgia
Lanifibranor; pan-PPAR agonist	10.1056/NEJMoa2036205	Patients with NASH	Group 1: lanifibranor 800 mg daily for 24 weeks (n = 93) Group 2: lanifibranor 1,200 mg daily for 24 weeks (n = 89) Group 3: placebo daily for 24 weeks (n = 92)	Decrease of at least 2 points in the SAF-A score without worsening of fibrosis: Significant, 55% vs. 33%, *P* = 0.007 (group 2 vs. group 3); No significant, 48% vs. 33%, *P* = 0.07 (group 1 vs. group 3)	Nausea, diarrhea, peripheral edema, anemia and weight gain
	10.1016/j.jhep.2024.12.045	Patients with type 2 diabetes and MASLD	Group 1: lanifibranor 800 mg for 24 weeks (n = 20) Group 2: placebo for 24 weeks (n = 18)	Change in IHTG (^1^H-MRS): Significant, full analysis set −44% vs. −12%; in completers −50% vs. −16%; both *P* < 0.01. (group 1 vs. group 2)	Weight gain

Abbreviations: IHTG, intrahepatic triglyceride content; TG, triglyceride; VLDL-TG, very low-density lipoprotein triglyceride; BMI, body mass index; T2DM, type 2 diabetes mellitus; ALT, alanine aminotransferase; AST, aspartate aminotransferase; MRI-PDFF, magnetic resonance imaging proton density fat fraction; LFC, liver fat content; NAFLD, non-alcoholic fatty liver disease; NAS, non-alcoholic fatty liver disease activity score; NASH, non-alcoholic steatohepatitis; MASLD, metabolic dysfunction-associated steatotic liver disease; 1H-MRS, proton magnetic resonance spectroscopy; EOT, end of treatment.

The combined use of PPAR agonists gives rise to clinical efficacy improvement theoretically, and dual- and pan-PPAR agonists have become the evolving frontier of NAFLD treatment. Accumulating evidence suggests that saroglitazar (a PPARα/γ agonist) and lanifibranor (a PPARα/β/γ agonist) are plausible treatments for NAFLD. The failure of elafibranor (a PPARα/δ agonist) to treat NAFLD implies that it would be better to apply dual PPAR agonists containing PPARγ agonists to achieve a more probable success in NAFLD treatment.

Despite the promise, numerous challenges remain. First, the heterogeneity of NAFLD progression requires personalized management. Accurate evaluation of metabolism, inflammation, fibrosis and comorbidities facilitates a better and more comprehensive understanding of disease status, which would be a critical variable to evaluate the feasibility of PPAR agonists. Second, long-term efficacy and safety data are still lacking. Most current published RCTs do not have the treatment durations greater than 3 years; hence, it is not possible to obtain long-term safety data. Under such circumstances, the anti-fibrotic effect of PPAR agonists is overlooked, as NAFLD is a chronic and progressive disease. Finally, the mechanism of NAFLD has not been elucidated, and other targets, such as THR-β and GLP-1, could become the alliance of PPAR combating NAFLD. The combined application of PPARs and other medications is worth studying to maximize clinical benefits. Although the use of clouds is still important for the treatment of NAFLD, a glimmer of hope from the use of PPAR agonists has appeared, and more studies are needed to move these clouds finally.
